# Moral Sensitivity, Empathy and Prosocial Behavior: Implications for Humanization of Nursing Care

**DOI:** 10.3390/ijerph17238914

**Published:** 2020-11-30

**Authors:** Iván Suazo, María del Carmen Pérez-Fuentes, María del Mar Molero Jurado, África Martos Martínez, María del Mar Simón Márquez, Ana Belén Barragán Martín, Maria Sisto, José Jesús Gázquez Linares

**Affiliations:** 1 Facultad de Ciencias de la Salud, Universidad Autónoma de Chile, Providencia 7500912, Chile; ivan.suazo@uautonoma.cl; 2 Department of Psychology, Faculty of Psychology, University of Almería, 04120 Almería, Spain; mpf421@ual.es (M.d.C.P.-F.); mmj130@ual.es (M.d.M.M.J.); amm521@ual.es (Á.M.M.); msm112@ual.es (M.d.M.S.M.); abm410@ual.es (A.B.B.M.); ms168@ual.es (M.S.); 3 Department of Psychology, Faculty of Psychology, Universidad Politécnica y Artística del Paraguay, Asunción 1628, Paraguay

**Keywords:** humanization, moral sensitivity, empathy, prosocial behavior, nursing care

## Abstract

Humanization of nursing is related to certain social and moral variables. Moral sensitivity, empathy, and prosocial behavior help understand a situation and make decisions that benefit the patient. The objective of this study is to find out how these variables are related, and define the differences in moral sensitivity, empathy, and prosocial behavior in humanization of nursing. We also analyzed the mediating role of empathy in the relationship between moral sensitivity and prosocial behavior. The sample was made up of 330 Spanish nurses aged 22 to 56, who completed the HUMAS Scale and adapted versions of the Basic Empathy Scale, the Moral Sensitivity Questionnaire, and the Prosocial Behavior Scale. Descriptive analyses, bivariate correlations and multiple mediation models were calculated. The results found significantly different mean scores between all the groups in responsibility and moral strength, cognitive empathy, and prosocial behavior, and in moral burden, the differences were in the high-humanization-score group compared to the low-score group. Furthermore, the mediation models showed the mediating effect of cognitive empathy between the responsibility, strength, and moral burden factors on prosocial behavior, but not of affective empathy. The study concluded that humanization in nursing is closely related to moral sensitivity, cognitive empathy, and prosocial behavior. This facilitates a helping, caring, and understanding attitude toward patient needs, but without the affective flooding that affective empathy can lead to.

## 1. Introduction

Nurses are indispensable in patient care and attention. The characteristics of their work demand strong physical and psychological involvement, which at the same time, if excessive or not managed adequately, may have negative consequences on their own health. This study was designed mainly to clarify the relationship of certain social and moral variables and their dimensions in the humanization of nursing as a contribution to the wellbeing, quality of life, and professional performance of nurses.

In the scope of healthcare, humanization refers to the personal competencies that enable professionals to do their jobs, respecting all human beings and attending to their physical, mental, and emotional care. It is, therefore, a multifactorial construct made up of cognitive and affective aspects that enable the nurse to work from a holistic, integral approach to attention and care [1]. On this basis, humanization of nursing care is related to certain social and moral variables.

### 1.1. Moral Sensitivity in Nursing Practice

Caring is an inner good of the healthcare professional, especially nurses. Humanized attention means ethical care, which enables them to understand patient needs beyond the purely physical. Therefore, these professionals assume amoral commitment with those they care for, which is demonstrated through attention to the patient, watching over their wellbeing and applying technical knowhow [2].

In this regard, we believe, as do other authors [3,4], that a lack of moral sensitivity in clinical practice could place the quality of professional healthcare performance at risk. Logically, high-quality clinical practice requires not only technical training and command of technological advances, but also congruent moral reasoning based on their own moral principles to guide them in their professional performance [5,6]. Therefore, to ensure quality attention in health, nurses must be conveyed and made aware of the code of ethics of daily clinical practice [7,8].

Although moral sensitivity has been conceptualized from different approaches, some authors suggest the need to advance in a concept of commonly delimited sensitivity that unambiguously orients and directs professional practice [9]. Rest [3] believes moral sensitivity to be the awareness of how one’s actions can affect others, making the nurse aware of moral problems that could arise while caring for others. Similarly, Ersoy and Göz [10] think moral sensitivity is the ability to recognize an ethical problem, while Weaver et al. [11] define moral sensitivity as a type of practical wisdom in making the patient feel comfortable, as well as professional satisfaction with attention given the patient.

For Lützén et al. [12], moral sensitivity in nursing is understanding the patient’s vulnerability and being aware of the moral implications of one’s decisions in any given situation. From this perspective, it would be personal predisposition that guides decision-making, involving an emotional response through a cognitive process that guides moral action and can involve moral tensions. Based on this conception, these authors designed an instrument, which to date is the most widely used by researchers to measure moral sensitivity, the Moral Sensitivity Questionnaire (MSQ) [13]. The brief version of this questionnaire evaluates three aspects of the construct: strength, responsibility, and moral burden.

Moral responsibility implies awareness of moral duties and obligations, and recognizing them when the professional, keeping in mind the individual patient’s viewpoint, is in a moral dilemma. Thus, moral responsibility makes patient attention and care possible, because it is directed toward others and contributes to action. Moral strength refers to moral resistance to adversity and valor or courage in making decisions and acting to benefit the patient instead of adopting a defensive position that inhibits action. Moral burden is produced by a problem or situation in which there is a discrepancy between moral values of professionals and patients that can generate negative feelings of stress, frustration, or guilt. These three dimensions are closely related to each other, and act together in moral sensitivity, which involves cognitive, affective, and moral capabilities [13].

### 1.2. Empathy and Prosocial Behavior in Nurses: Relationship with Moral Sensitivity

Moral sensitivity has been positively related to prosocial behavior and empathy [14,15,16]. The first of these variables, moral sensitivity, is one of the essential components guiding adaptive prosocial behavior patterns [17]. Prosocial behavior is understood as voluntary behavior directed at benefiting others [18]. Individuals more sensitive to moral problems, usually make prosocial decisions [19]. Therefore, healthcare professionals with more moral sensitivity are more capable of appreciating situations that pose a moral dilemma and making prosocial decisions directed at benefiting the patient. The importance of reinforcing such behavior in humanized care has also been emphasized [20,21]. In this regard, Ruttan and Lucas [22] mention that prioritizing a search for monetary profit instead of humanized treatment in employment is associated with less prosocial behavior and with moral disconnection.

Prosociability is a multidimensional construct [23], which, in addition to the behavior mentioned above, has an affective dimension including empathy, as a tendency to understand and share the emotional states of others.

Concerning empathy in nurses, problems in managing demands and concerns related to the patient’s values could negatively affect the quality of their professional performance. Nevertheless, some findings agree that empathy improves clinical practice [24,25,26]. Empathy is considered one of the moral sentiments [17]. According to Hoffmann’s [15,27] moral socialization theory, empathy is interpreting cognitively what is happening to another person, but also feeling appropriately in that situation. Hoffman defines empathy as being able to feel how others feel, placing oneself in their affective position, such that the emotional connection propitiates prosocial behavior toward them [27]. Based on this definition, Jollife and Farrington [28], operationalized this construct in an instrument called the Basic Empathy Scale (BES), which evaluates these two components of empathy as cognitive empathy (“knowing” what the other feels) and affective empathy (“feeling” in a manner congruent with what the other person feels). Other researchers have also found a relationship between emotional regulation and this factorial division of empathy [29]. Oliveira et al. [30] suggested that empathy, in addition to the cognitive and affective factors, involves emotion regulation mechanisms. Thus, when emotional activation triggered by exposure to the feelings of others is not managed properly, mechanisms regulating emotional responsiveness could have a key role in the empathic process by eliminating overburden and emotional distress [31].

Empathy facilitates team work and patient-centered care given by healthcare professionals [24,25] and is related to subjective wellbeing [32]. Some studies have emphasized the value of empathy in humanized care [33,34,35,36,37,38], especially cognitive empathy, which has been established as a variable directly involved in humanization of care in nursing [39]. It also has an important role in reducing exhaustion [40,41,42], improving the wellbeing of healthcare professionals [43]. Some authors have found an association between moral sensitivity and empathy in nursing [44,45]. For example, Jo and Kim [46] found a moderate positive correlation between cognitive empathy and moral sensitivity and lower between moral sensitivity and affective empathy. In line with this, Wong [47] mentions that healthcare professionals should show cognitive and affective empathy, along with moral sensitivity to be able to cope with the distress and suffering of others. When these individual variables are aligned, they promote effective, humanized healthcare.

### 1.3. The Present Study

The role of nurses is essential in optimizing both health of the population and the patient care experience [48,49]. To increase the knowledge on the individual characteristics involved in the humanized action beneficial to the patient could be useful in improving and wellbeing of the workers themselves. Keeping in mind the empirical findings mentioned, the following hypotheses were posed: both moral sensitivity and empathy are positively associated with prosocial behavior in nursing (H1). Similarly, humanization in nursing has a close relationship with high levels of moral sensitivity, empathy, and prosocial behavior (H2). In particular, we expected to find significant differences in each of the dimensions of these variables (moral sensitivity, empathy, and prosocial behavior) depending on the level of nurses’ humanization. So professionals with higher scores in humanization would have higher levels of moral sensitivity. This requires high cognitive empathy to facilitate perspective-taking and prosocial action, while not flooding the professional–patient relationship (H3). Lastly, attempting to further explore the role of empathy in moral development and prosocial behavior, and following Hoffman’s theory [15,27], the relationship between moral sensitivity and prosocial behavior was analyzed. Building on the assumption that cognitive empathy would exert a stronger mediating effect than affective empathy, particularly in the relationship between moral sensitivity and prosocial behavior of nurses.

Therefore, the objective of this study is to establish the relationships between these variables, defining the differences in moral sensitivity, empathy, and prosocial behavior based on humanization in nursing. We also wanted to analyze the mediating role of empathy in the relationship between moral sensitivity and prosocial behavior.

## 2. Methods

### 2.1. Participants

The original sample of this descriptive cross-sectional study consisted of 338 Spanish nurses, of whom those who answered incongruently or at random, as found by control questions distributed at random in the questionnaires, were eliminated. This answer control system was based on questions with only one obviously correct answer, such as, “I am answering a survey right now”. Eight such cases were eliminated because of errors in questions of this kind. Therefore, the final sample was made up of a total of 330 Spanish nurses with a mean age of 32.30 (*SD* = 7.54) in a range of 22 to 56. The participants were 83.9% (*n* = 277) women and 16.1% men, with a mean age of 32.62 (*SD* = 7.92) and 30.62 (*SD* = 4.90), respectively. Distribution of the professionals by marital status was 60.9% (*n* = 201) single, 36.4% (*n* = 120) married, 2.4% (*n* = 8) separated or divorced, and 0.3% widowed (*n* = 1).

Sample inclusion criteria were that participants must be practicing nurses, that is working at the time of data acquisition. Their employment status by specific contract type was 63.9% (*n* = 211) limited-time or temporary contract, and the remaining 36.1% (*n* = 113) permanent or stable contract.

### 2.2. Instruments

*Moral Sensitivity Questionnaire-Revised Version (MSQ-R).* The brief version designed by Lützen et al. [13], consists of nine items with six answer choices, where 1 = strongly disagree and 6 = strongly agree. Its application procures information on three dimensions of moral sensitivity: sense of moral burden (“My ability to sense the patient’s needs means that I do more than I have the strength for”), moral strength (“My ability to sense the patient’s needs is always helpful in my work”) and moral responsibility (“I always feel a responsibility for the patient receiving good care even if the resources are inadequate”). It was recently validated with a sample of Brazilian nurses, showing a reliability coefficient with Cronbach’s alpha of 0.82 [50]. In this study, the reliability indices were optimum, both for the global moral sensitivity scale (α = 0.84, ω = 0.87), and the moral strength dimension (*α* = 0.88, *ω* = 0.89). The reliability indices for the moral burden dimensions were considered acceptable (*α* = 0.65, *ω* = 0.66), while reliability was somewhat lower in the moral responsibility dimension (*α* = 0.53, *ω* = 0.54).

Basic Empathy Scale (BES). This was developed by Jollife and Farrington [28] to measure the cognitive and affective empathy. The brief version, translated into Spanish by Oliva et al. [51] and validated by Merino-Soto and Grimaldo-Muchotrigo [52], consists of a nine-item scale with five answer choices, where 1 = strongly disagree and 5 = strongly agree. It provides a score on affective empathy (“After being with a friend who is sad for some reason, I usually feel sad”) and another on cognitive empathy (“When someone is depressed, I usually understand how they feel”). Previous studies have found high internal consistency in the general scale and its dimensions [28,51,52]. In this study, the internal consistency indices showed optimum values for the global scale (α = 0.87, ω = 0.88), α = 0.85 and ω = 0.86 on the affective empathy subscale and α = 0.90 and ω = 0.91 on the cognitive empathy subscale.

Prosocial Behavior scale (PBS; [29]). This scale consists of 16 items that differentiate between more and less prosocial behavior. The answers are coded on a five-choice Likert scale which goes from 1 = never to 5 = always/almost always. The authors found high internal consistency with a Cronbach’s alpha of 0.91. In this study, the indices were also optimum, both for the general prosocial behavior scale (α = 0.93, ω = 0.94) and for the prosocial behavior dimensions (“I try to help others”) (α = 0.90, ω = 0.91) and empathy and social support (“I connect with the mood of someone who is suffering”; “I try to console someone who is sad”) (α = 0.80, ω = 0.81).

Healthcare Professional Humanization Scale (HUMAS). This scale was developed by Pérez et al. [39] with a sample of nurses. It consists of 19 items that evaluate five dimensions: disposition to optimism (“I look forward the future enthusiastically”), sociability (“When I take care of my patients, I try to put myself in their place”), emotional understanding (“When someone treats me badly, I try to understand the reasons and keep trying to treat that person well”), self-efficacy (“I am able to differentiate the changes in others’ moods and act accordingly”), and affection (“When I perform my professional labor, I usually feel distressed”). The answers are rated on a five-choice Likert-type scale from 1 = never to 5 = always. The McDonald’s Omega coefficients found in this study were optimum, both for the general scale (*ω* = 0.88) and for its respective dimensions: *ω* = 0.86 in disposition to optimism, *ω* = 0.86 in sociability, *ω* = 0.88 for emotional understanding, *ω* = 0.86 for self-efficacy, and *ω* = 0.89 in affection.

### 2.3. Procedure

Before collecting the data, the participants were assured that data processing in the study would comply with the applicable standards of data security, confidentiality, and ethics. They were specifically told that the principles of the Helsinki Declaration (1964): respect for the individual, the right to self-determination and decision-making, use of informed consent, wellbeing of the subject above the interests of research, etc., would be followed. In addition to these ethical standards, the study complied with Spanish Organic Law 3/2018 of December 5th, on Protection of Personal Information and guaranteeing digital rights in effect in the country where the research was carried out (Spain) [53], guaranteeing online questionnaire completion.

The study was approved by the University of Almería Bioethics Committee (Ref: UALBIO2019/30) (Spain). Data were collected in a CAWI survey (Computer Aided Web Interviewing). The survey was distributed over the social networks (snowball sampling). Participation was voluntary and on the first page, before answering the questionnaire, the participants were given information on the study and its purpose, where they had to mark a box indicating their informed consent before they could start taking the survey. A series of control questions were included to monitor for random or incongruent responses, and those subjects were removed from the sample.

### 2.4. Data Analysis

First, a Pearson’s bivariate correlation analysis was performed to examine the relationships between variables, and an ANOVA and the corresponding post- hoc tests were applied to determine whether there were any differences between HUMAS groups with regard to mean scores on moral sensitivity, empathy, and prosocial behavior. The η^2^ and ω^2^ coefficients were used for the effect size. To find the “HUMAS group” variable, the total score was recorded by applying the cutoff scores proposed by the authors of the questionnaire (0–73 = Low, 74–81 = Medium, 82–95 = High).

Then, to determine the mediating effect of empathy and of the behavior of each of its components (affective empathy vs. cognitive empathy) in the relationship between moral sensitivity and prosocial behavior, a multiple mediation analysis was performed. The PROCESS macro for SPSS [54] with 5000 bootstrap samples was used to compute the models.

The McDonald’s Omega coefficient [55] was estimated to examine the reliability of the instruments used for data collection, following the proposal and guidelines of Ventura-León and Caycho [56].

## 3. Results

### 3.1. Correlations and Descriptive Statistics

Table 1 shows the positive relationships between the components of moral sensitivity (responsibility, strength, and moral burden) and the two empathy factors (affective and cognitive), where the cognitive component of empathy was the one with the strongest correlations.

The humanization construct was positively correlated with responsibility, strength, and sense of moral burden. Sense of moral burden showed the weakest correlation.

Prosocial behavior was positively correlated with all three moral sensitivity factors and both empathy factors. However, the strongest correlations were with responsibility, moral strength, and cognitive empathy.

Finally, the total HUMAS score was positively correlated with all the variables analyzed except affective empathy, with which there was no statistically significant relationship.

As observed in Table 2, the mean scores on the moral sensitivity and prosocial behavior variables differed between the groups formed by their general HUMAS factor scores (0–73 = Low, 74–81 = Medium, 82–95 = High). In empathy, only the cognitive factor differed between the HUMAS groups.

Post-hoc tests (Bonferroni) extracted significant differences between the groups in responsibility and moral strength, in cognitive empathy and in prosocial behavior. In moral burden, the differences were between the high-score HUMAS group and the low-score group.

### 3.2. Mediation of Empathy in the Relationship between Moral Sensitivity and Prosocial Behavior

**Figure 1:**
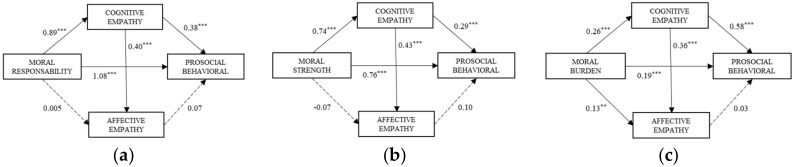
Statistical diagrams of the direct effects in the proposed mediation models. [Note: (**a**) moral responsibility and prosocial behavior; (**b**) moral strength and prosocial behavior; (**c**) moral burden and prosocial behavior. ** *p* < 0.01, *** *p* < 0.001].

One multiple-mediation model was proposed for each moral sensitivity factor, with two mediator variables, cognitive empathy (Mediator 1) and affective empathy (Mediator 2). In all cases, prosocial behavior was the dependent variable (Figure 1).

Model (a): A significant effect of moral responsibility on cognitive empathy *β* = 0.89, 95% CI (0.70, 1.08) was observed, but not on affective empathy *β* = 0.005, 95% CI (−0.20, 0.21). The effect of the mediators on the dependent variable was significant for cognitive empathy *β* = 0.38, 95% CI (0.27, 0.49), but not for affective empathy *β* = 0.07, 95% CI (−0.03, 0.17). The direct effect of moral responsibility on prosocial behavior was significant *β* = 1.08, 95% CI (0.87, 1.28).

Model (b): There was a significant effect in the relationship between moral strength and cognitive empathy *β* = 0.74, 95% CI (0.62, 0.86), but not affective empathy *β* = −0.07, 95% CI (−0.22, 0.07). The relationships between the mediators and the dependent variable were found to be significant for cognitive empathy *β* = 0.29, 95% CI (0.17, 0.41), but this did not hold true for affective empathy *β* = 0.10, 95% CI (−0.00, 0.20). The direct effect of moral strength on prosocial behavior was significant *β* = 0.76, 95% CI (0.61, 0.91).

Model (c): Significant direct effects were observed in the relationship between sense of moral burden and cognitive empathy *β* = 0.26, 95% CI (0.17, 0.36) and with affective empathy *β* = 0.13, 95% CI (0.04, 0.22). The effect of the mediators on the dependent variable was significant for cognitive empathy *β* = 0.58, 95% CI (0.46, 0.70), but not for affective empathy *β* = 0.03, 95% CI (−0.08, 0.15). The direct effect of moral responsibility on prosocial behavior was significant *β* = 0.19, 95% CI (0.08, 0.29).

The results of analysis of indirect effects using bootstrapping for each model (Table 3) were the following:

Model (a): The effect of the total model was *β* = 1.45, 95% CI (1.25, 1.65), with an R^2^ = 0.38. Indirect effects through Path IE_1_ (moral responsibility → cognitive empathy → prosocial behavior) were significant *β* = 0.34, 95% CI (0.23, 0.47).

Model (b): The total effect of the model was *β* = 1.01, 95% CI (0.88, 1.14), with an R^2^ = 0.41. Of the indirect effects, Path IE_1_ (moral strength → cognitive empathy → prosocial behavior) was significant *β* = 0.21, 95% CI (0.13, 0.31). Path IE_2_ (moral strength → cognitive empathy → affective empathy → prosocial behavior), although significant, had a low coefficient *β* = 0.03, 95% CI (0.00, 0.07).

Model (c): The total effect of the model was *β* = 0.35, 95% IC (0.24, 0.46), with an R^2^ = 0.10. Of the indirect effects, Path IE_1_ (moral burden → cognitive empathy → prosocial behavior) was significant *β* = 0.15, 95% CI (0.08, 0.23).

## 4. Discussion

In the first place, the results of the correlation analysis showed that both moral sensitivity and empathy were positively associated with prosocial behavior, confirming our first research hypothesis. However, our data stressed certain dimensions of moral sensitivity and empathy. Thus, the higher the nurses’ scores in moral responsibility and moral strength, the higher their scores in prosocial behavior were. Moreover, as nurses’ scores in cognitive empathy rose, prosocial behavior scores also were higher. In the relationship between moral sensitivity and empathy, the moral sensitivity dimensions, as mainly cognitive variables, were also correlated more strongly with cognitive empathy than affective empathy. Similar results were also found by Jo and Kim [46] in their study with a sample of Korean nurses, where moral sensitivity correlated moderately with cognitive empathy and had a weak correlation with affective empathy.

With regard to the second study hypothesis, the finding that the moral strength and responsibility dimensions, in this order of priority, had the strongest relationship with personal competence in humanization in the relationship between humanization and moral sensitivity, is especially important. However, moral burden had very little correlation with humanization of nurses. Weaver et al. [11] found that stress and anxiety negatively influenced the solution of ethical dilemmas. Likewise, as mentioned by Ersoy and Göz [10], nurses are sensitive to questions of confidentiality, telling the truth and charity, but are less sensitive when dealing with the right to reject treatment, and so how they should act should be delimited, focusing decision-making on moral conflict. Their moral distress can also be lessened by eliminating the contributing factors. At the very least, these findings are cause for reflection about the analysis of moral sensitivity and its relationship with other variables, to determine how it can be strengthened. It might also be mentioned that humanization was associated with more prosocial behavior in the nurses’ repertoire, with positive consequences for the patient.

Our findings showed that there is a moderate relationship between humanization and cognitive empathy, but not with affective empathy. One possible interpretation could be that empathy contributes to humanization in nursing when it is present in prudent doses, and that cognitive empathy contributes more to humanized treatment than affective empathy. Three aspects of humanization are clearly related to affective achievements: emotional understanding (or ability to experience, understand, and manage emotions effectively), self-efficacy (or the ability to manage complicated, stressful situations successfully and appropriately), and affection (or vulnerability to the existence of a maladjustment of expectations between what professionals think is part of their responsibility in acting and how the patient expects professionals to act) [1,39]. There should be a balance between all these components of humanization for the professional to show prosocial behavior, and these results may demonstrate that higher levels of cognitive empathy enable situations to be interpreted more objectively. That is, without strong emotional involvement, thereby contributing to more effective decision-making by healthcare professionals. Other studies have found results supporting these interpretations. Caprara and Steca [29] validated a structural model in which a strong sense of efficacy in regulating positive and negative affect was associated with high perceived efficacy in managing social relations and in empathic commitment with the emotional experiences of others. In this study, interpersonal self-efficacy directly affected prosocial behavior and fully mediated influence of affective self-efficacy on it.

Moreover, our findings on differences in the moral sensitivity, prosocial conduct, and empathy factors by level of humanization confirmed our third hypothesis. Specifically, the results showed that professionals with low scores on the HUMAS had significantly lower scores on moral burden, responsibility and strength, cognitive empathy and prosocial behavior. On the contrary, workers in the group with high humanization capacity had significantly higher scores on these factors. In the case of professionals with medium humanization capacity, the scores were lower than those of the workers in the high HUMAS group, but higher than those in the low HUMAS group on all the above factors, except moral burden. In this dimension of moral sensitivity, there were no significant differences from the other two groups. In line with this, Wong [47] states that, to promote effective humanized healthcare practices, the moral sensitivity and empathy factors must be high. Furthermore, it should be mentioned that there were no significant differences in affective empathy in any of the groups. Previous studies have already noted a stronger connection of cognitive empathy than affective with humanization of care [46]. It is therefore not surprising that there were no differences in affective empathy by humanization level.

Finally, our fourth hypothesis, in which cognitive empathy would have a mediating effect on the relationship between moral sensitivity and prosocial behavior, was verified. Such that cognitive empathy acted as a buffer variable in the effect of moral sensitivity on prosocial behavior. Our data previously showed high correlations between cognitive empathy and moral strength and responsibility. Like so low correlations between both dimensions of moral sensitivity and affective empathy, where the relationship between affective empathy and moral burden was moderate. Therefore, cognitive empathy most explained the indirect relationship between prosocial behavior and moral strength (41% of the variability found), moral responsibility (38%) and moral burden (10%), in this order. These findings shed light on the role of empathy in moral development and prosocial behavior proposed in the Hoffman theory [15,27]. They particularly identify a less outstanding role of affective empathy in humanization of nurses, considering the high correlation between cognitive empathy and prosocial behavior. The latter could also explain the attitude of helping, caring for, and understanding patient needs.

Before concluding, the limitations of any cross-sectional study should be mentioned, and we propose the suitability of longitudinal designs in future studies on the subject. Self-report measures used to evaluate the variables (humanization, moral sensitivity, empathy, and prosocial behavior) are very useful for studies with large samples, because application and data processing are economical, but they could be completed with observational measures and participant interviews, among other methods of collecting information. We should also mention that the unit where the nurses were working was not taken into consideration. The functions and type of relationship and contact with patients is different in the different healthcare units, and therefore, in future, it would be advisable to include this variable in the analysis. Finally, in future studies, it would also be recommendable to include other variables, such as age or years of experience that could be affecting the performance of these professionals from a social and moral perspective.

## 5. Conclusions

Humanization in nursing, which includes cognitive, affective, and social skills, has a close relationship with moral sensitivity, in particular with certain aspects, such as moral strength and responsibility, and to a lesser extent, with moral burden. This moral sensitivity factor is associated with concerns and moral dilemmas, product of disagreement between the patients’ own desires and the obligations and duties of professionals, generating stress and feelings of guilt in the nurses.

Furthermore, affective empathy had a weak relationship with humanization. In fact, no differences were found between the various groups set up based on humanization scores. This shows that affective empathy exerts a secondary role in nursing care compared to the more outstanding role of cognitive empathy. Professionals with high affective and low cognitive empathy could become flooded by the patient’s emotional state, unable to manage such emotions. On the contrary, a high level of cognitive empathy exerts a positive effect on patient care, by improving the quality of the relationship through an understanding of their emotions and needs.

At the same time, the mediation models confirmed that moral sensitivity exerted an indirect effect on the prosocial behavior, which was mediated by cognitive empathy. Our analyses showed that cognitive empathy largely explained the indirect relationship between moral sensitivity and prosocial behavior, especially in moral strength and responsibility. These findings lead us to reflect on the analysis of this construct, and in particular, on how it can be strengthened. Probably, emotional education would enable nurses to be more sensitive to individual patient needs, generating moral action, developing humanization, and contributing to a greater extent to moral, empathic, and humanized action. Organizations could also give employee training programs for stimulating and conveying ethical and moral values in caring for others. This, along with training in the ability to understand how the patient feels, would increase prosocial behavior of healthcare professionals, in other words, increase behaviors seeking the benefit of the patient, thereby improving the quality of care, as well as wellbeing of these workers.

In conclusion, we should emphasize humanization of nursing as a vehicle for wellbeing, quality of life, and optimum professional performance. Not only does it orient toward improving the quality of life of nurses, but can also benefit care quality, and therefore, the general health of patients, and lastly, adequate functioning of the healthcare system and policies.

## Figures and Tables

**Table 1:** Bivariate correlation matrix and descriptive statistics.

		MR		MS		MB		AE		CE		PB		HUMAS
MR	Pearson’s r	—												
	Upper 95% CI	—												
	Lower 95% CI	—												
MS	Pearson’s r	0.718	***	—										
	Upper 95% CI	0.767		—										
	Lower 95% CI	0.661		—										
MB	Pearson’s r	0.546	***	0.530	***	—								
	Upper 95% CI	0.617		0.604		—								
	Lower 95% CI	0.465		0.448		—								
AE	Pearson’s r	0.191	***	0.197	***	0.262	***	—						
	Upper 95% CI	0.293		0.298		0.360		—						
	Lower 95% CI	0.085		0.090		0.159		—						
CE	Pearson’s r	0.450	***	0.562	***	0.290	***	0.420	***	—				
	Upper 95% CI	0.532		0.632		0.386		0.505		—				
	Lower 95% CI	0.359		0.484		0.188		0.327		—				
PB	Pearson’s r	0.618	***	0.643	***	0.327	***	0.283	***	0.557	***	—		
	Upper 95% CI	0.681		0.702		0.420		0.380		0.628		—		
	Lower 95% CI	0.547		0.575		0.227		0.181		0.478		—		
HUMAS	Pearson’s r	0.517	***	0.580	***	0.226	***	0.024		0.432	***	0.599	***	—
	Upper 95% CI	0.592		0.647		0.326		0.132		0.516		0.664		—
	Lower 95% CI	0.433		0.503		0.121		−0.084		0.340		0.525		—
	*M*	9.95		15.01		17.09		13.74		19.59		25.52		77.45
	*SD*	1.54		2.30		3.34		2.97		3.07		3.62		8.24

Note. MR = moral responsibility; MS = moral strength; MB = moral burden; AE = affective empathy; CE = cognitive empathy; PB = prosocial behavior. HUMAS = humanization. *** *p* < 0.001.

**Table 2:** Moral sensitivity, empathy and prosocial behavior by HUMAS group. Descriptive statistics and ANOVA.

HUMAS	Moral Responsibility			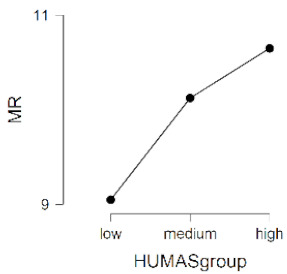
	*n*	*M*	*SD*	
High	100	10.65	1.26	
Medium	129	10.12	1.19	
Low	101	9.05	1.75	
*F* = 33.93, *p* < 0.001 (η^2^ = 0.17, ω^2^ = 0.16)				
HUMAS	Moral Strength			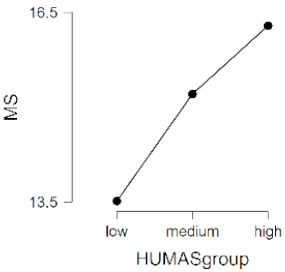
	*n*	*M*	*SD*	
High	100	16.29	1.66	
Medium	129	15.20	1.90	
Low	101	13.51	2.48	
*F* = 47.62, *p* < 0.001 (η^2^ = 0.23, ω^2^ = 0.22)				
HUMAS	Moral Burden			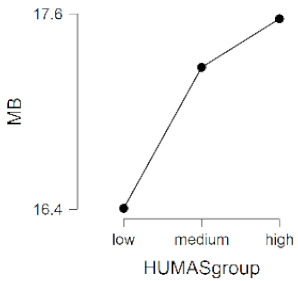
	*n*	*M*	*SD*	
High	100	17.57	3.42	
Medium	129	17.27	3.36	
Low	101	16.40	3.14	
*F* = 3.38, *p* < 0.05 (η^2^ = 0.02, ω^2^ = 0.01)				
HUMAS	Affective empathy			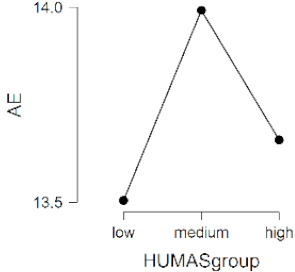
	*n*	*M*	*SD*	
High	100	13.66	3.44	
Medium	129	13.99	2.75	
Low	101	13.50	2.73	
*F* = 0.81, *p* = 0.444				
HUMAS	Cognitive empathy			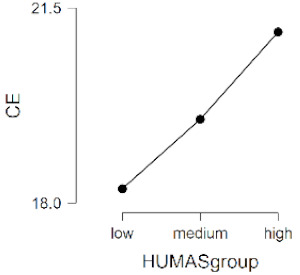
	*n*	*M*	*SD*	
High	100	21.07	2.98	
Medium	129	19.50	2.69	
Low	101	18.25	2.99	
*F* = 24.14, *p* < 0.001 (η^2^ = 0.13, ω^2^ = 0.12)				
HUMAS	Prosocial Behavior			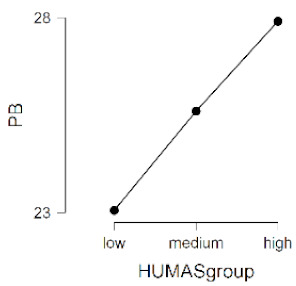
	*n*	*M*	*SD*	
High	100	27.91	2.53	
Medium	129	25.60	2.56	
Low	101	23.05	4.08	
*F* = 61.58, *p* < 0.001 (η^2^ = 0.27, ω^2^ = 0.26)				

Note. MR = moral responsibility; MS = moral strength; MB = moral burden; AE = affective empathy; CE = cognitive empathy; PB = prosocial behavior. HUMAS = humanization.

**Table 3:** Direct, total, and indirect effects.

(a) Moral responsibility and Prosocial behavior	β	SE	t	95% CI
Direct effect: Moral responsibility → Prosocial behavior	1.08 ***	0.10	10.32	(0.87, 1.28)
Total effect: Moral responsibility → Prosocial behavior	1.45 ***	0.10	14.24	(1.25, 1.65)
IE 1: Moral responsibility → Cognitive empathy → Prosocial behavior	0.34	0.05		(0.23, 0.47)
IE 2: Moral responsibility → Cognitive empathy → Affective empathy → Prosocial behavior	0.02	0.02		(−0.01, 0.07)
IE 3: Moral responsibility → Affective empathy → Prosocial behavior	0.00	0.00		(−0.01, 0.02)
**(b) Moral strength and Prosocial behavior**	**β**	**SE**	**t**	**95% CI**
Direct effect: Moral strength → Prosocial behavior	0.76 ***	0.07	10.02	(0.61, 0.91)
Total effect: Moral strength → Prosocial behavior	1.01 ***	0.06	15.21	(0.88, 1.14)
IE 1: Moral strength → Cognitive empathy → Prosocial behavior	0.21	0.04		(0.13, 0.31)
IE 2: Moral strength → Cognitive empathy → Affective empathy → Prosocial behavior	0.03	0.01		(0.00, 0.07)
IE 3: Moral strength → Affective empathy → Prosocial behavior	−0.00	0.00		(−0.03, 0.00)
**(c) Moral burden and Prosocial behavior**	**β**	**SE**	**t**	**95% CI**
Direct effect: Moral burden → Prosocial behavior	0.19 ***	0.05	3.70	(0.08, 0.29)
Total effect: Moral burden → Prosocial behavior	0.35 ***	0.05	6.27	(0.24, 0.46)
IE 1: Moral burden → Cognitive empathy → Prosocial behavior	0.15	0.03		(0.08, 0.23)
IE 2: Moral burden → Cognitive empathy → Affective empathy → Prosocial behavior	0.00	0.00		(−0.00, 0.01)
IE 3: Moral burden → Affective empathy → Prosocial behavior	0.00	0.00		(−0.01, 0.02)

Note. IE = indirect effect, SE = standard error, CI = confidence interval. Sample size bootstrap for indirect effects = 5000; β = non-standardized regression coefficient; *** *p* < 0.001.
